# Identification of putative fecundity-related gustatory receptor genes in the brown planthopper *Nilaparvata lugens*

**DOI:** 10.1186/s12864-018-5391-5

**Published:** 2018-12-27

**Authors:** Kui Kang, Pan Yang, Li-E Chen, Rui Pang, Lu-Jun Yu, Wen-Wu Zhou, Zeng-Rong Zhu, Wen-Qing Zhang

**Affiliations:** 1 State Key Laboratory of Biocontrol and School of Life Sciences, Sun Yat-sen University, Guangzhou, 510275 Guangdong China; 2 State Key Laboratory of Rice Biology, Institute of Insect Sciences, Zhejiang University, Hangzhou, 310029 Zhejiang China

**Keywords:** *Nilaparvata lugens*, Gustatory receptor, Alternative splicing, Fecundity

## Abstract

**Background:**

The insect gustatory system plays a central role in the regulation of multiple physiological behaviors and the co-evolution between insects and their hosts. The gustatory receptors (Gr) are important to allow insects to sense their environment. It is critical to the selection of foods, mates and oviposition sites of insects. In this study, the *Gr* family genes of the brown planthopper (BPH) *Nilaparvata lugens* Stål (Hemiptera: Delphacidae) were identified and analyzed, and their potential relationship to the fecundity of BPH was explored by RNA interference (RNAi).

**Results:**

We identified 32 putative *Gr* genes by analyzing transcriptome and genome data from BPH. Most of these Gr proteins have the typical structure of seven transmembrane domains. The BPH *Gr* genes (*NlGrs*) were expressed in virtually all tissues and stages, whilst higher transcript accumulations were found in adult stages and in the midguts of females. Based on the phylogenic analysis, we classified *NlGrs* into five potential categories, including 2 sugar receptors, 2 Gr43a-like receptors, 7 CO_2_ receptors, 5 bitter receptors and 13 *NlGrs* with unknown functions. Moreover, we found that 10 *NlGrs* have at least two alternative splicing variants, and obtained alternative splicing isoforms of 5 *NlGrs*. Finally, RNAi of 29 *NlGrs* showed that 27 of them are related to the transcript levels of two fecundity related genes vitellogenin and vitellogenin receptor.

**Conclusions:**

We found 32 *Gr* genes in BPH, among which at least 27 are required for normal expression of fecundity markers of this insect pest. These findings provide the basis for the functional study of *Grs* and the exploration of potential genes involved in the monophagous character of BPH.

**Electronic supplementary material:**

The online version of this article (10.1186/s12864-018-5391-5) contains supplementary material, which is available to authorized users.

## Background

Insects interact with their environment primarily through a sensitive chemosensory system that can detect and discriminate a diverse array of chemicals. This system plays critical roles in the survival and reproductive success of insects, mediating their behavioral responses to food, mates, and oviposition sites [1, 2]. Gustatory receptors (Grs), members of the chemosensory superfamily are mainly distributed in the chemosensory organs. They are responsible for distinguishing CO_2_ and non-volatile chemicals including the nutrients and toxins [3].

Insect Grs, with a typical seven transmembrane structure, were first identified in the *Drosophila melanogaster* genome based on a bioinformatic approach [4]. Further studies found that there are 68 gustatory receptor proteins in *D. melanogaster*, encoded by 60 gustatory receptor genes through alternative splicing [5–7]. Most gustatory receptor proteins are extraordinarily divergent, sharing only 8–12% sequence identity at the amino acid level. Some of this divergence could improve the diversity of Grs’ responses to ligands [7]. Based on the known ligands to which they respond, Grs were grouped into sugar receptors, CO_2_ receptors, Gr43a-like receptors, bitter receptors, sex pheromone receptors, and unknown receptors [8–13]. With the development of genome sequencing in insects, insect Gr genes are identified in an increasing number of additional species: *Anopheles gambiae* has 52 Gr genes encoding 76 Gr proteins [14], and *Aedes aegypti* has 79 Gr genes encoding 114 Gr proteins [15]. *Bombyx mori* and *Tribolium castaneum* have 65 and 220 Gr genes, respectively [16, 17]. *Helicoverpa armigera* showed the second highest number (197) of Gr genes among all insect species studied [18].

Alternative pre-mRNA splicing greatly expands the proteome diversity within species by creating different combinations of exons from the same genomic loci [19], and has been most extensively studied in *D. melanogaster* [20]. Among sixty *D. melanogaster* Grs, three genes have alternatively spliced transcripts. *Gr23a* and *Gr39a* encoded two and four predicted proteins, respectively [4], and *Gr28b* encodes five predicted proteins [7]. Gr39a is a multifunctional receptor set, and alternative splicing is the mechanism for the generation of molecular forms responsible for different functions, such as pheromonal perception and host plant selection [21]. *Gr28b.d*, one alternatively spliced transcript of *Gr28b*, encoded a potential warmth sensor (Gr28B(D)) [22]. *T. castaneum* Gr214 is a massive alternatively spliced locus with 30 alternative long 5′ exons spliced into three shared 3′ exons encoding the C terminus [16]. The alternative splicing pattern of gustatory receptor has also been found in other insect species, such as *A. aegypti* [15] and *B. mori* [17]. However, the function of these alternatively spliced forms remains unclear.

The brown planthopper (BPH) *Nilaparvata lugens* (Hemiptera: Delphacidae) is one of the most devastating insect pests of rice [23]. Vitellogenin (*Vg*) and vitellogenin receptor (*VgR*) were always used as molecular markers of fecundity [24].Gustatory receptors may play an important role in the interaction of BPH and rice by detecting chemical compounds in rice. In this study, we identified 32 *Grs* in BPH based on transcriptome and genome data. Then we analyzed the alternative splicing patterns of all *NlGrs*, and found that 10 of them had alternative splicing events; further studies showed that alternative splicing could alter the protein structure of some *NlGrs*. Finally, we employed the RNA interference (RNAi) experiments and found that 27 *NlGrs* are required for normal expression of fecundity markers of this insect pest.

## Results

### Transcriptome sequencing and sequence assembly

We performed next-generation sequencing on a cDNA library constructed from the adult head, leg and midgut of BPH. All clean reads were aligned to the reference transcripts (generated in our laboratory, unpublished) and genome [25] of BPH, and identified approximately 47,000 genes with an N50 length of 2300 bp (see Additional file 1: Table S1). The mapping rate was approximately 75%, except in the midgut (57.9%), which contains a large number of enteric microorganisms (see Additional file 1: Table S1).

### Annotation and identification of gr genes

To identify as many gustatory receptors as possible, three strategies were used. Firstly, we obtained 33 and 10 potential *Gr* sequences from the transcriptome data and genome data, respectively, using reciprocal hit of tBLASTn. 36 potential *Gr* sequences were identified after combining these sequences. The length of them was between 216 bp and 3921 bp, and most sequences were shorter than 1000 bp (see Additional file 2: Table S2). We then used pfam to build the second structure model of GR, PF02949, PF08395, and PF06151 and searched for the target sequences in the protein database. Using this method, we obtained 20 potential GR protein sequences, and 3 were identified as olfactory receptors via blast in NCBI; 13 were covered with previously potential sequences. After removing the sequence whose predicted protein length are less than 100 amino acids, only one new potential GR sequence was found, XLOC_018226:3–81 (see Additional file 3: Table S3). Finally, using a conserved sequences search, we obtained 21 potential GR protein sequences, all of which were covered with previously sequences. In summary, we obtained 37 potential *Gr* sequences using bioinformatic analyses.

To further determine whether these genes are *Gr* genes, we used 3′ rapid amplification of cDNA ends (3’ RACE) and 5’ RACE to obtain longer fragments. After sequencing, the result was tested by using BLAST and compared with the database of National Center for Biotechnology Information. The results showed that 6 potential sequences were not *Grs* and 4 were identical to each other. In conclusion, we identified 27 *Grs* in BPH. By combining the sequences with 10 previously reported gustatory receptors [25], a total of 32 *NlGrs* were identified. Detailed information of the identified and cloned *Grs* in BPH is available (Table 1).

**Table 1:** Nucleotide, amino acid, scaffold distribution, alternative splice variant and other information for the identified *NlGrs*

GENE	TMHMM	TMPred	HMMTOP	AA	Nucleic acids(bp)	Scaffold	Predicted alternative splice variant number
*NlGr1*	8	8	8	436	1554	KN153269.1	0
*NlGr2*	7	8	9	461	1717	KN153837.1	2
*NlGr3*	7	8	8	440	1631	KN153837.1	0
*NlGr4*	4	4	4	265	798	KN153837.1	0
*NlGr5*	6	7	7	425	1670	KN152938.1	0
*NlGr6*	5	5	5	488	2188	KN152682.1	10
*NlGr7*	8	8	8	413	1605	KN153186.1	0
*NlGr8*	5	6	6	357	2176	KN153141.1	10
*NlGr9*	4	6	4	400	3216		10
*NlGr10*	7	8	8	471	2121	KN151984.1	2
*NlGr11*	7	7	8	466	1342	KN152173.1	0
*NlGr12*	6	6	6	339^a^	1785	KN152706.1	0
*NlGr13*	8	8	7	401	1625	KN152706.1	0
*NlGr14*	6	6	7	339	1536	KN152506.1	0
*NlGr15*	4	4	6	321	1604	KN152468.1	2
*NlGr16*	4	8	6	383	1149	KN152007.1	0
*NlGr17*	7	8	9	520	1561	KN152004.1	4
*NlGr18*	3	7	6	374	1308	KN153751.1	3
*NlGr19*	3	3	3	218^a^	1730	KN152376.1	4
*NlGr20*	6	7	7	449	2062	KN152775.1	2
*NlGr21*	7	6	8	352	1435	KN154548.1	0
*NlGr22*	6	6	5	370	1110	KN154035.1	0
*NlGr23*	7	7	6	398	1537	KN153338.1	2
*NlGr24*	7	7	7	379	1141	KN152091.1	0
*NlGr25*	3	3	3	218	1580	KN152147.1	0
*NlGr26*	5	5	5	429	1750	KN152989.1	3
*NlGr27*	0	0	0	0^b^	212		0
*NlGr28*	6	6	6	357	1071	KN154570.1	2
*NlGr29*	6	6	6	373	2032	KN152059.1	0
*NlGr30*	0	0	0	0^b^	416		0
*NlGr31*	3	3	3	153	1926	KN152885.1	0
*NlGr32*	0	0	0	131	394	KN154495.1	0

^a^Has more than one ORF regions, and the longest ORF was used for protein translation

^b^ORF is incomplete

### Phylogenetic analysis

**Fig. 1:**
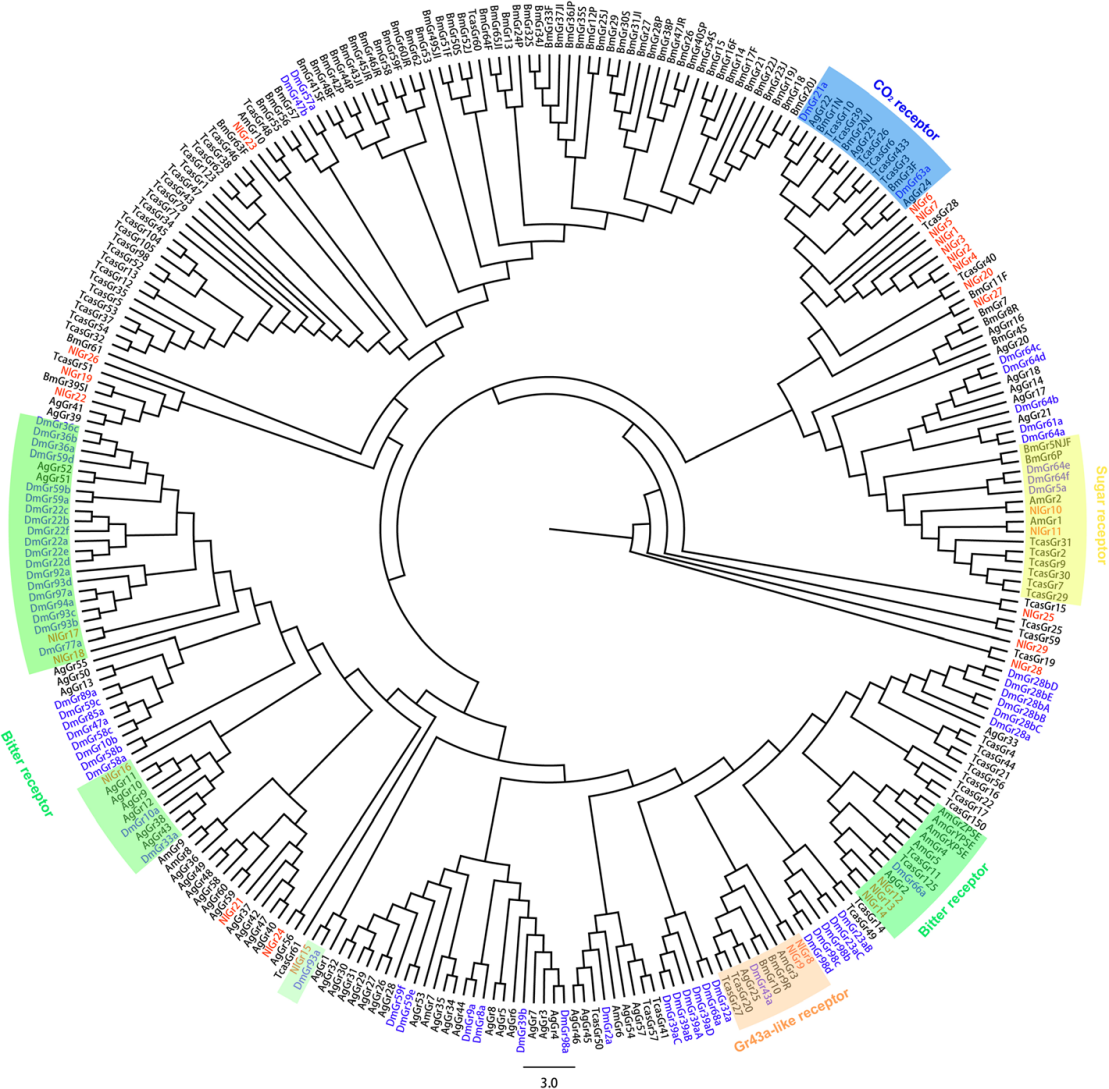
Phylogenetic relationship of 29 *Nilaparvata lugens* gustatory receptors (*NlGrs*) and five published insect gustatory receptors. The reference sequence includes 68 *Drosophila melanogaster* Grs (DmGrs), 65 *Bombyx mori* Grs (BmGrs), 13 *Apis mellifera* Grs (AmGrs), 58 *Aedes aegypti* Grs (AgGrs), 62 *Tribolium castaneum* Grs (TcasGrs). *Dm*Grs and *Nl*Grs are highlighted with blue and red letters, respectively. The maximum-likelihood tree was calculated using default settings and the Jones-Taylor-Tornton (JTT) model with partial deletions and 2000 bootstrap replications

The phylogenetic analysis result revealed that *NlGr1–7* are potential members of the CO_2_ receptor subfamily (Fig. 1); *NlGr8* and *NlGr9* are potential members of the Gr43a-like (*Drosophila*) receptor subfamily [13]. *NlGr10* and *NlGr11* are potential members of the insect sugar receptor subfamily [26, 27]. *NlGr12–NlGr14* are orthologs to the *DmGr66a* protein which is responsible for the recognition of various bitter compounds [3]. In addition, *NlGr15*-*NlGr16* might belong to the narrowly tuned Grs required for some bitter compounds, as they are in the same branch as *D*mGr93a or *DmGr33a* [28]. Moreover, *NlGr17*- *NlGr18* have a simple orthologous relationships with *DmGr77a* and other *DmGrs*, whilst their functions remain unclear [29]. The remaining 11 *NlGrs* do not have confident phylogenetic relationships with Grs from other insects (Fig. 1).

### Expression profiles of *NlGr* genes

**Fig. 2:**
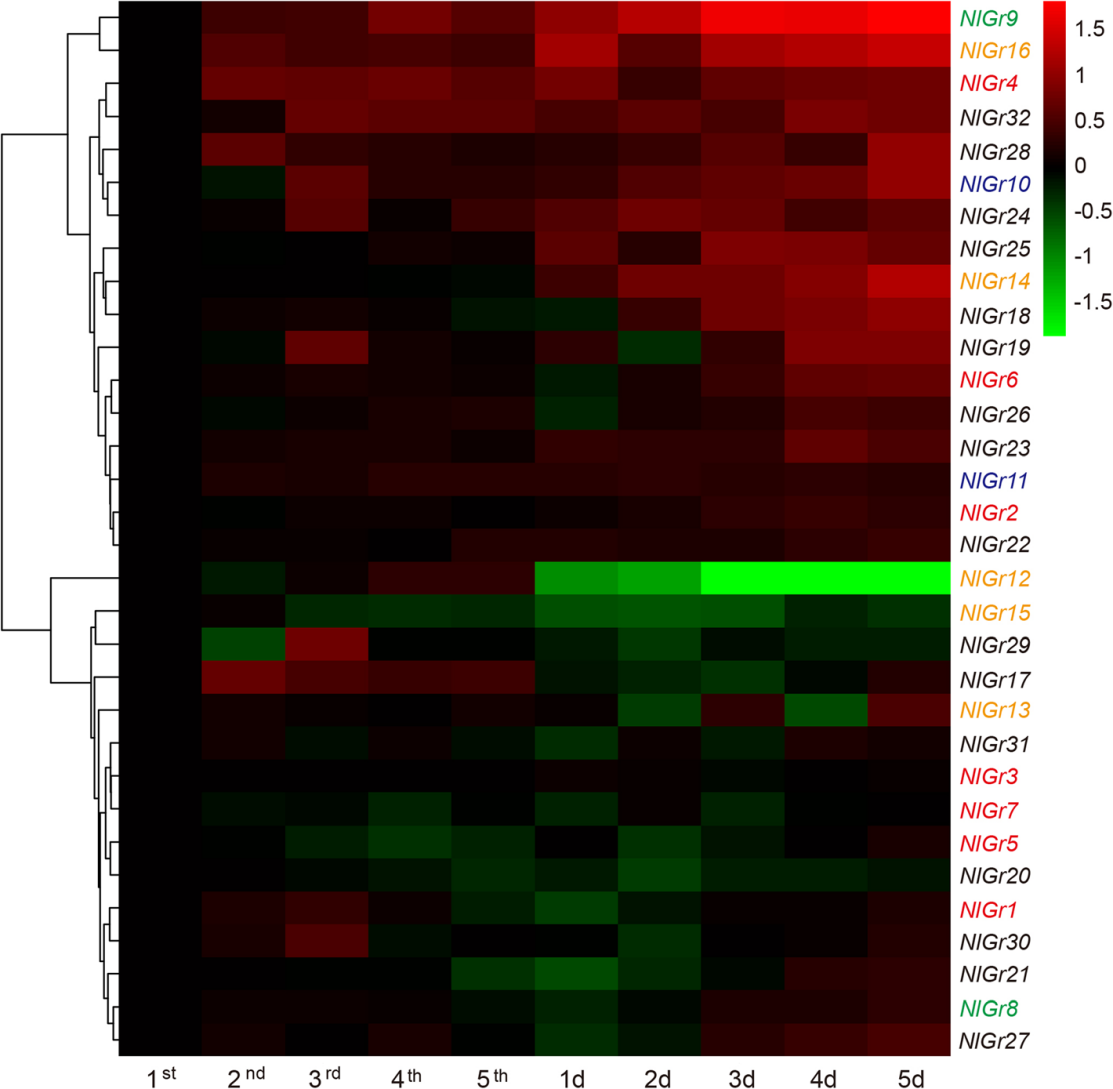
The expression pattern of *NlGrs* in different developmental stage. *NlGrs* expression at various stages including 1st-instar nymph (1st), 2nd-instar nymph (2nd), 3rd-instar nymph (3rd), 4th-instar nymph (4th), 5th-instar nymph (5th), and 1-day- to 5-day-old female adults (1d-5d); mRNA levels of 1st-instar nymphs were used as controls. Different color fonts represent different predicted subfamilies: red: CO_2_ receptor subfamily; green: Gr43a-like receptor subfamily; blue: sugar receptor subfamily; orange: bitter receptor subfamily; black: unknown functions receptor. All mRNA levels are normalized by the *β-actin* mRNA levels, and data are shown as the mean (*n* = 3)

**Fig. 3:**
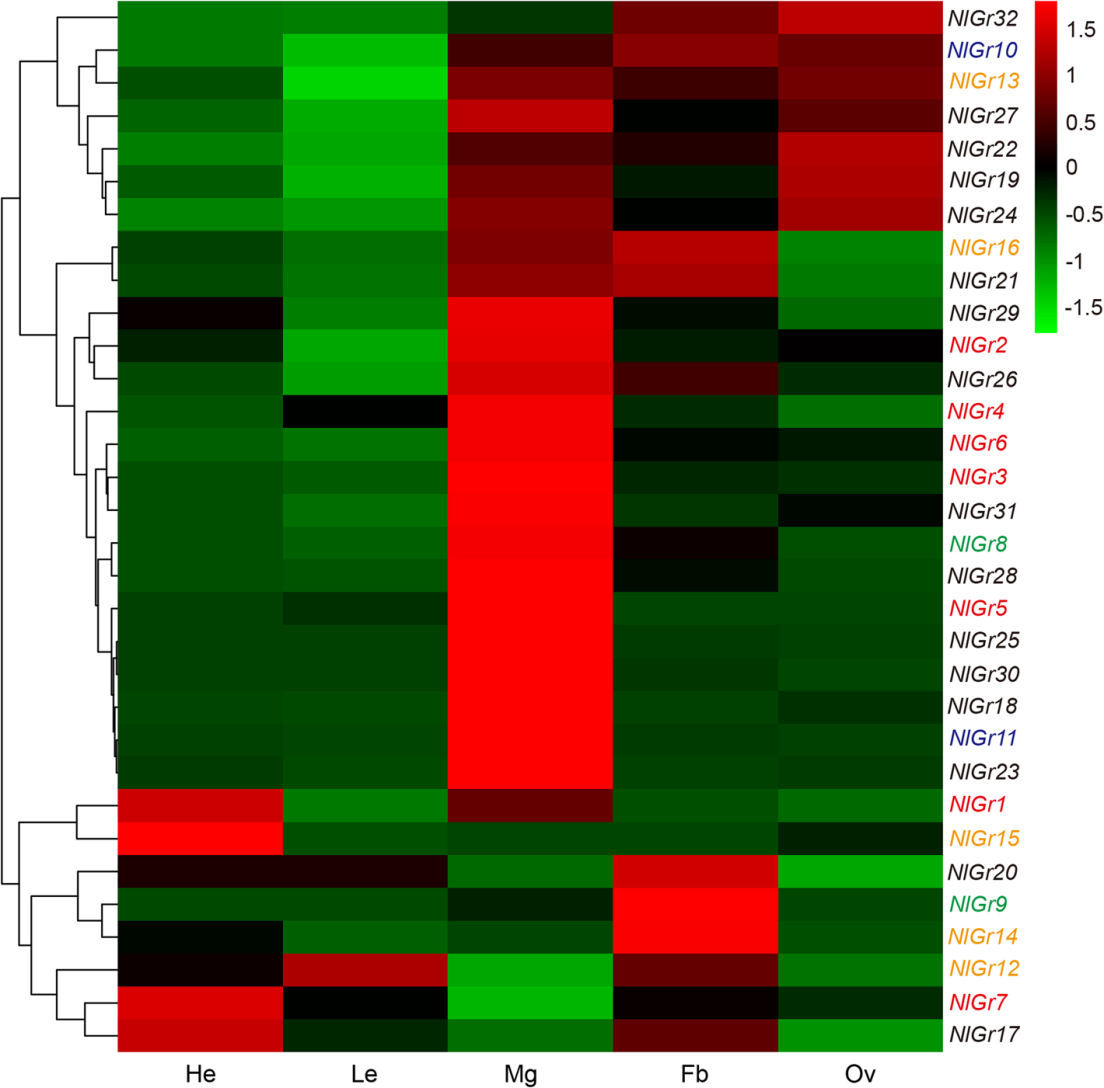
Tissue specific expression pattern of *NlGrs*. Expression of *NlGrs* in various female tissues including the heads (He), legs (Le), midgut (Mg), fat body (Fb) and ovaries (Ov); mRNA levels of legs were used as controls. Different color fonts represent different predicted subfamilies: red: CO_2_ receptor subfamily; green: Gr43a-like receptor subfamily; blue: sugar receptor subfamily; orange: bitter receptor subfamily; black: unknown functions receptor. All mRNA levels are normalized by the *β-actin* mRNA levels. Data are shown as the mean (n = 3)

qRT-PCR was used to investigate the expression pattern of the *Gr* genes in different tissues and developmental stages of BPH. Across the developmental stages, some Grs have a higher transcript accumulation in 4th or 5th day old brachypterous female adult than other developmental stages, such as *NlGr6, NlGr9*, *NlGr10, NlGr14, NlGr18, NlGr19, NlGr16* and *NlGr25* (Fig. 2). Five Grs, including *NlGr1, NlGr12, NlGr15, NlGr17, and NlGr20*, have a higher mRNA expression in nymph stages (Fig. 2). In particular, *NlGr12* was only detected in nymph stages, and *NlGr29* showed the highest expression in 3rd instars nymphs. Moreover, all *NlGr* genes were found in the heads, legs, midgut, fat body or female ovaries (Fig. 3). 21 *NlGr* genes were expressed in multiple tissues. For example, among them, *NlGr24* and *NlGr32* were mainly detected in 3 tissues: the midgut, fat body and female ovaries. *NlGr16* and *NlGr21* were expressed in the midgut and fat body. Conversely, 11 *NlGr* genes were mainly expressed in specific tissues. For example, *NlGr5, NlGr11, NlGr18, NlGr23, NlGr25, NlGr28, NlGr30,* and *NlGr31* were most highly expressed in the midgut. *NlGr9* and *NlGr14* were mainly expressed in the fat body. And *NlGr15* was mainly expressed in the adult heads (Fig. 3). By analyzing the DEG data, the expression profiles of *NlGrs* in head, midgut and legs were similar to the qRT-PCR results.

### Alternative splicing analysis of *NlGrs*

**Fig. 4:**
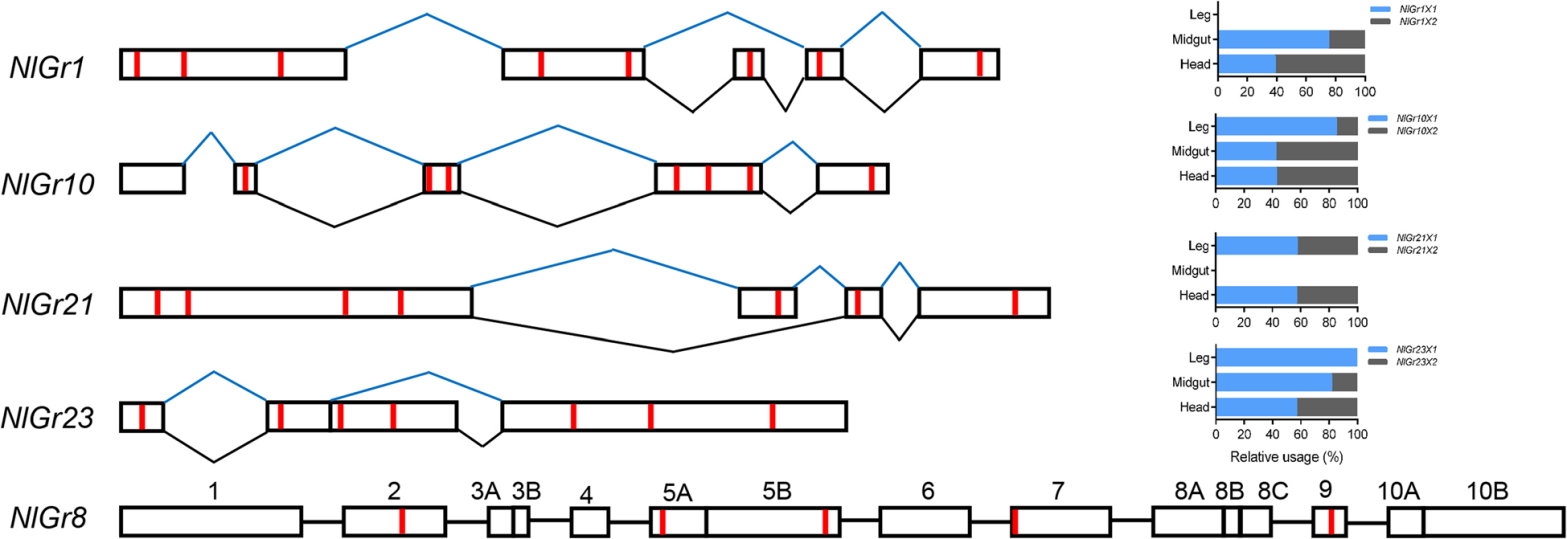
Alternatively spliced pattern of five *NlGrs*. The data were verified by PCR with genomic DNA and cDNA as templetes. Bars represent exon sequence; lines represent intron sequences. Blue lines and black lines represent different splicing modes. Red lines in bars represent transmembrance domain. Splicing patterns include cassette exon skipping (*NlGr1, NlGr21*), alternative first exons (*NlGr10*), and alternative 5ʹ splice site (*NlGr23*). The histograms showed the different isoform relative usage from the RNA-seq data, blue bars represent blue line spliceosome

**Fig. 5:**
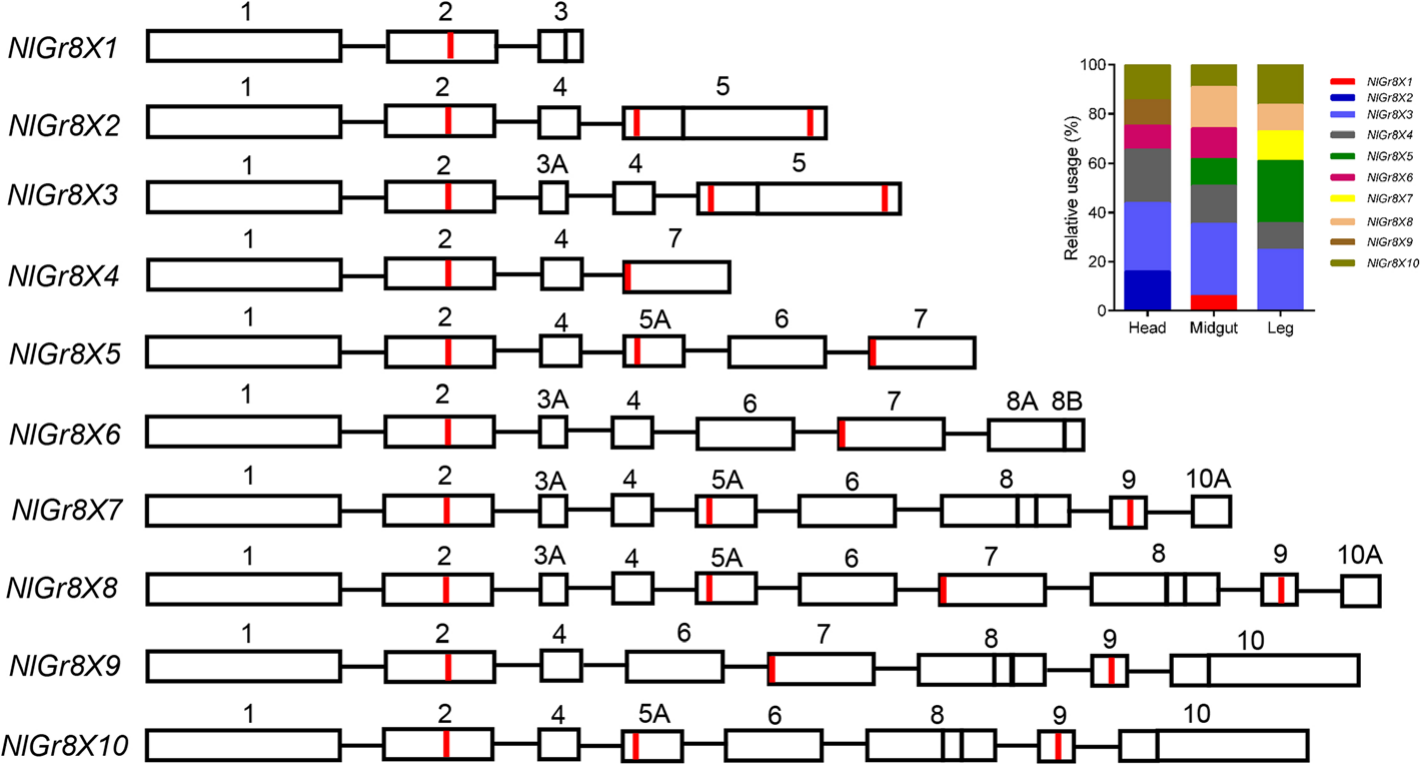
Ten alternatively spliced patterns of *NlGr8.* Bars represent exon sequence, and lines represent intron sequence. The data were verified by PCR with genomic DNA and cDNA. Red lines in bars represent transmembrance domain. Splicing patterns include cassette exon skipping, alternative 3ʹ splice site, alternative last exons and mutually exclusive cassettes. The histograms showed the different isoform relative usage from the RNA-seq data, different color bars represent different spliceosome

By assembling the transcriptome, a total of 70,361 transcripts were obtained. Among them, 49,930 transcripts were found to contain multiple exons, and 27,880 alternative splicing events were identified. Among the 32 *NlGr* genes, 13 genes have potential alternative splicing events (Table 1). We verified all of the predicted alternative splicing events for *NlGrs* by PCR, and found that 10 of them had at least two splicing variants (Table 2). By comparison with the genome of BPH, we obtained alternative splicing forms of five *NlGrs*: *NlGr1, NlGr8, NlGr10, NlGr21,* and *NlGr23* (Figs. 4 and 5)*.*

**Table 2:** Clone of alternatively splicing of *NlGrs* in *Nilaparvata lugens*

Gene	Type	Predicted number of alternative splice variants	Verified number of alternative splice variants
*NlGr1*	CO_2_ receptor	0	2
*NlGr8*	Gr43a-like receptor	10	10
*NlGr10*	Sugar receptor	2	2
*NlGr12*	Bitter receptor	0	2
*NlGr19*	Unknown	4	3
*NlGr21*	Unknown	0	2
*NlGr23*	Unknown	2	2
*NlGr25*	Unknown	0	3
*NlGr26*	Unknown	3	2
*NlGr31*	Unknown	0	3

*NlGr10*, a putative sugar receptor, had an alternative first exon event, and was found to have two splicing variants: *NlGr10a*, which encodes 470 amino acids; and *NlGr10b*, which encodes 458 amino acids. *NlGr1* and *NlGr21* showed cassette exon skipping events, and were found to have two splicing variants, respectively. Another unknown receptor, *NlGr23* had an alternative 5′ splice site, and the splice site was located in its second exon. *NlGr8*, with ten exons and ten splicing forms, had the most complex splicing events among all the *NlGrs* (Fig. 5). The alternative splicing may change the protein structure of these 5 *NlGrs*. For example, one splicing variants of *NlGr23* lost the second and third transmembrane domains, and the *NlGr10* splice variants generated by the alternative first exon and encoded an N-terminal altered NlGR10 protein.

### Effects of dsRNA injection on *NlVg* and *NlVgR* expression

**Fig. 6:**
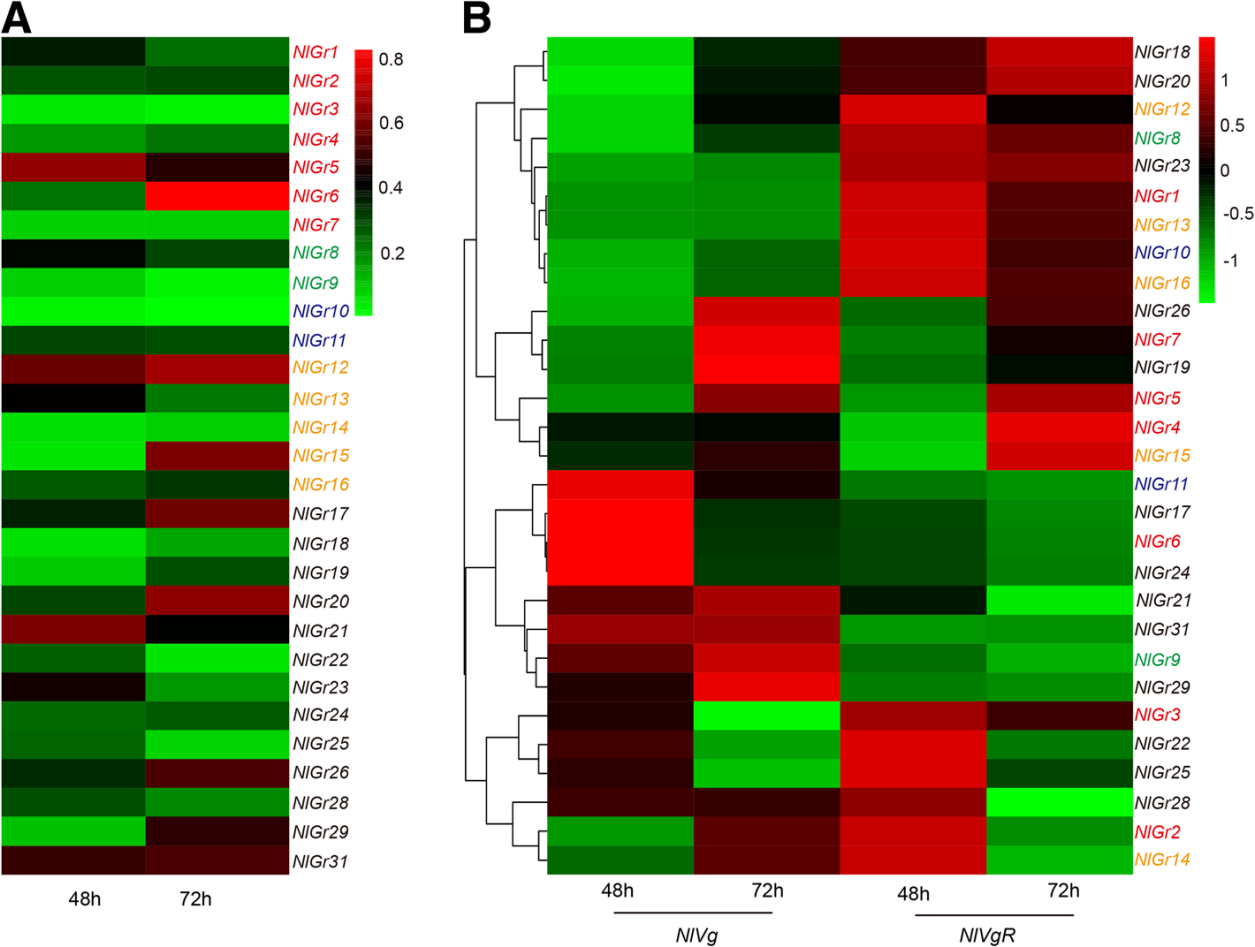
Knockdown the expression of *NlGrs* in BPH and its effect on *NlVg* and *NlVgR* transcript accumulation. **a**: mRNA level of target genes; **b**: mRNA level of vitellogenin (*Vg*) and vitellogenin receptor (*VgR*) was detected by qRT-PCR; mRNA levels of females treated with dsGFP were used as controls. Different color fonts represent different predicted subfamilies: red: CO_2_ receptor subfamily; green: Gr43a-like receptor subfamily; blue: sugar receptor subfamily; orange: bitter receptor subfamily; black: unknown functions receptor. All mRNA levels are normalized by the *β-actin* mRNA levels and data are shown as the mean (n = 3)

To further investigate the function of the *NlGr* genes in BPH, RNAi experiments were performed. Gene silencing of the target genes was achieved by dsRNA injection (Fig. 6a, see Additional file 4: Table S4), except for three *NlGrs* (*NlGr27, NlGr30* and *NlGr32*), whose nucleotide sequences were less than 450 bp and were too short for primer design. We found three patterns of *NlVg* and *NlVgR* mRNA levels in BPH after silencing the 29 *NlGrs* (Fig. 6b). In the first type, the transcript of *NlVg* and *NlVgR* was decreased when BPH was injected with ds*Nl*Gr. The genes with this pattern included 17 *NlGrs*: *NlGr1, NlGr3, NlGr5, NlGr6*, *NlGr7, NlGr8, NlGr10, NlGr12, NlGr13, NlGr14*, *NlGr15, NlGr17, NlGr18, NlGr20*, *NlGr22, NlGr25, and NlGr29*. In the second type, the transcript of *NlVg* was increased, while it was decreased for *NlVgR*, when BPH was injected with ds*NlGr19*, ds*NlGr28* or ds*NlGr31*. And the third type, either *NlVg* or *NlVgR* had changed mRNA level after injection. For example, the *NlVgR* expression was decreased when treated with ds*NlGr2,* ds*NlGr9* or ds*NlGr11*, whilst no significant change in *NlVg* mRNA level were found; and the *NlVgR*, but not *NlVg*, showed increased mRNA level when BPH was treated with ds*NlGr4* or ds*NlGr16* (Fig. 6b). Furthermore, *NlGr23* and *NlGr26* did not appeare to have significant effect on the expression of *NlVgR*, while the *NlVg* expression was significantly decreased or increased at 72 h after with the injection of them, respectively. In summary, except for *NlGr21* and *NlGr24*, the other tested 27 *NlGrs* affected the transcript accumulation of *NlVg* or *NlVgR*. These results implied that *NlGrs* may play an important role in modulating *NlVg* or *NlVgR* expression in BPH. Since *Vg* and *VgR* were always used as molecular markers of fecundity [24], it is likely that these 27 *NlGrs* are required for normal expression of fecundity markers.

### *NlGr25* response to resistant rice varieties

**Fig. 7:**
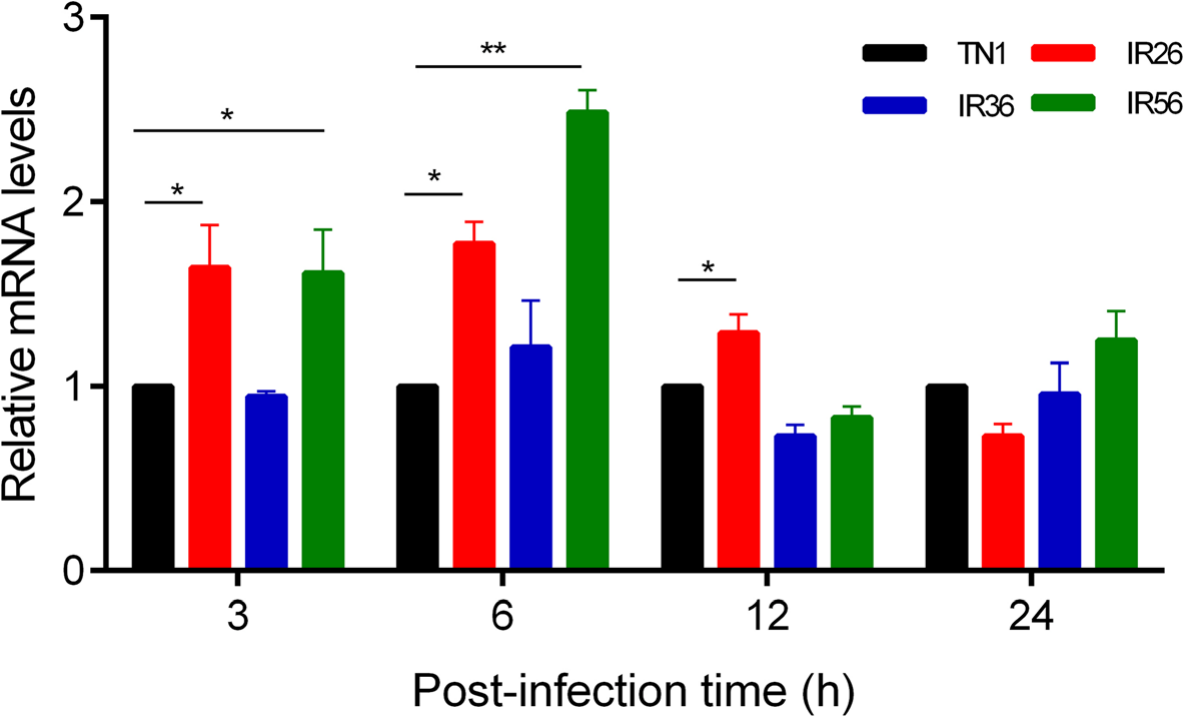
Expression of *NlGr25* induced by various rice cultivars. *NlGr25* mRNA abundance was measured by quantitative real-time PCR. Total RNA was extracted from BPH at 3, 6, 12 and 24 h after feeding from different rice cultivars. TN1: Taichung Native 1, a BPH susceptible variety; IR36: International Rice 36, a BPH-resistant variety contains resistance gene *bph2*; IR56: International Rice 56, a BPH-resistant variety contains resistance gene *Bph3*. Data are shown as the means ± SE of three replicates. Significant differences between two treatments are marked with an asterisk (*t*-test), *, at the 0.05 level; **, at the 0.01 level

qRT-PCR was used to measure the mRNA levels of *NlGr25* from the whole body fed on the sensitive rice variety (TN1) and resistance rice varieties (IR26, IR36, IR56). The results showed that *NlGr25* transcript levels were significantly increased at 3 h, 6 h and 12 h post fed on IR26 compared to fed on TN1. Similar results were observed for IR56. Whereas, feeding on IR36 didn’t influence expression of *NlGr25* in BPH (Fig. 7).

## Discussion

We identified 32 *Gr* genes in BPH by combining transcriptome and genome data [25]. Insect *Gr* families appear to be adapted to their ecological niche. For example, the honeybee *Apis mellifera* has an expansion of the olfactory receptor gene family (163 Ors) and only 10 *Grs* [30]. It has been hypothesized that bees have a limited need for *Grs* in plant secondary metabolite discrimination due to their typical foraging and social behavior [30]. *Helicoverpa armigera* has three times as many *Grs* (197) than *Ors* (64), which may be linked to this species’ capacity as a successful generalist, as the expansion presumably broadens the range of plant secondary metabolites detected by this species [18]. The number of *NlGr* was less than that in the aphid *Acyrthosiphon pisum* (72 *Grs*) [31], which is also a hemipteran insect. This may be because BPH is a monophagous insect that only feeds on rice, possibly explaining the reduced number of *Gr* genes. *A. pisum* is a type of omnivorous agricultural pest, and more *Grs* may allow it to recognize more secondary or primary metabolites from different host plants. Based on phylogenetic analysis, we classified 29 *NlGrs* into five categories: sugar receptors, Gr43a-like receptors, CO_2_ receptors, bitter receptors and Grs with unknown functions. No loss of *NlGr* function shows that BPH *Grs* may be able to detect and discriminate large amount of metabolites in rice. Rapid changes in the “bitter” receptor repertoire have been described in *Drosophila* flies, apparently concomitant with changes in their plant hosts [32].

Transcriptome and qRT-PCR approaches were used to analyze the expression profile of *NlGr* genes in different tissues and at different stages, and the results showed that *NlGrs* was expressed almost in all tissues and stages. It is similar to the observations in *Nasonia vitripennis* whose *Grs* are most likely expressed in diverse chemosensory tissues, such as mouthparts, legs, and the ovipositor, as well as the digestive tract [33]. In other insects, such as *T. castaneum*, the *Grs* are mainly expressed in the labium and maxillae, femur, tibia, and tarsus of the adult prolegs [34]. In *Aedge aegypti*, the gustatory sensilla are located in stereotyped positions on the labella and tarsi of the legs [35], and there is also specific expression of *Grs* in these tissues [36]. *Dm*Gr genes are found in abdominal tissues and the taste organs, including the labellum, pharyngeal organs, and tarsi [37, 38]. Since BPH is a piercing sucking insect, the midgut is the main tissue used to digest food, absorb nutrients and remove toxic substances. *NlGr2*5 was most highly expressed in the midgut and in 3th day female adults, we propose that that it might affect behaviors of BPH by responding to metabolite(s) in rice. Similarly, 12 *DmGrs* were expressed in midgut enteroendocrine cells, implying that the *Grs* have chemosensory roles in the intestine to regulate physiological functions, such as food uptake, nutrient absorption, or sugar homeostasis [38]. Some *DmGrs* have a higher mRNA level in the nymph stage, indicated that they play a role in the development of insects, as previously reported in *Drosophila* [39].

Alternative splicing is increasingly of interest in the study of evolution and adaptation of insects, as it provides the means for rapid functional innovations via very economical genomic changes [20]. However, to date, alternative splicing phenomenon has not been well studied for gustatory receptor family. In *D. melanogaster,* only three Gr genes (*Gr23a, Gr39a* and *Gr28b*) were found to have alternative splicing events [7], and alternative splicing in three genes brings the total number of Grs from 71 to 81 in *Drosophila suzukii* [40]. In BPH*,* 10 of 32 *NlGrs* have alternative splicing events, a higher percentage than that in *Drosophila*, which may be caused by the factor of the low number of *Gr* genes. Insect Grs often have a seven transmembrane structure [4], and the alternative splicing can change the transmembrane structure or the number units spanning the membrane and may greatly alter the function of the GR protein. The *DmGr39a* gene produces four isoforms through alternative splicing of various 5′-most exons [7]. The seventh transmembrane domain and C-terminus are encoded by the last three exons, which are shared by the four isoforms, while the N-terminus and the first six transmembrane domains, which are unique to each isoform, are encoded by four alternative 5′ exons [21]. The RNAi of *DmGr39a* reduced courtship levels toward females in *Drosophila*, whilst it is not clear whether each isoform has a different function [21]. *NlGr10* showed alternative first exon events, and the two isoforms both exhibited an entire seventh transmembrane domain. However, these two isoforms responded to different sugars (unpublished data). As a Gr43a-like receptor, *NlGr8* was verified to have as many as ten splicing forms. Its homologous gene *Dm*Gr43a plays a critical role in sensing internal fructose levels in the fly brain and is both necessary and sufficient to sense hemolymph fructose and promote feeding in hungry flies, while suppresses feeding in satiated flies [41]. Thus, we propose that *NlGr8* may play important roles in recognizing carbohydrates in rice phloem juice.

Gustatory receptors can affect insect behavior, such as feeding, oviposition or mating, by recognizing non-volatile plant secondary substances [3, 42]. The butterfly, *Papilio xuthus*, uses a gustatory receptor *Px*ut*Gr1* to select its host during oviposition [42]. *DmGr33a* is required for proper oviposition to avoid ovicidal coumarin-laced food in *D. melanogaster* [3]. *DmGr66a* in the ventral cibarial sensory organ (VCSO) mediates the egg-laying attraction to lobeline and in neurons on the legs mediates positional aversion [43]. Functional and behavioral studies showed that other Gr genes (*Dm*8a, *Dm*93a, *Dm*98b) responded to bitter compounds to induce avoidance responses, such as caffeine, umbelliferone, L-canavanine [28, 44]. Our results showed that *NlGr* can be induced by resistant rice varieties, suggesting that they may recognize some resistant or toxic metabolites in resistant rice, and subsequently affect BPH feeding behavior and oviposition behavior. And our previous study confirmed that *NlGr11*, a sugar receptor, accelerated the fecundity of BPH through the AMPK- and AKT-mediated signaling pathways [45].

## Conclusion

In conclusion, we discovered that *N. lugens* has 32 *Gr* genes, most of which belong to the bitter receptor and an unknown function clade. Ten of these Gr genes have two or more alternative splicing variants. The RNAi results showed that 27 *NlGrs* are required for normal expression of fecundity markers. These findings contribute to the understanding of the interactions between BPH and rice.

## Methods

### Insects

A *N. lugens* laboratory strain was originally obtained from Guangdong Academy of Agricultural Sciences (GDAAS; Guangdong, China), and this strain was reared in a continuous laboratory culture on BPH-susceptible rice plants (Huang Hua Zhan, bought from GDAAS). The insects were maintained in the laboratory at 26 ± 2 °C with 80 ± 10% humidity and a light-dark cycle of L16:D8h.

### Sample preparation

A total of 100 3-day-old brachypterous females were used for tissue dissection. The following tissues were used for RNAseq: head, midgut and legs. The fat body and ovary were also used for the gene expression profiling. Different development stages of BPH, from the first nymph to five days after emergence, were collected. Total RNA was isolated using the TRIzol method (Invitrogen, Carlsbad, CA, USA) according to the manufacturer’s protocol. The samples were treated with DNase, and their RNA contents were quantified using a microvolume spectrophotometer (NanoDrop 2000, Thermo Fisher Scientific, Waltham, MA, USA).

### DGE analysis

The cDNA libraries were constructed and sequenced by the Beijing Genomics Institute (BGI, Shenzhen, China) on the Illumina sequencing platform (HiSeq™ 2000), producing 100-bp pair-end reads (SRA accession: PRJNA504931). All clean reads were aligned to the reference transcript (generated in our laboratory, unpublished) of *N. lugens* using TopHat [46].

### Gene annotation

We used three methods to identify the BPH gustatory receptor by assembled transcripts. Firstly, we download all the insect gustatory receptor gene family from National Center for Biotechnology Information (https://www.ncbi.nlm.nih.gov/) as a database, and the basic local alignment was used to search the DGE data of head, midgut and legs, respectively. Then tBLASTn searches against the NCBI database to confirm that the obtained sequences belong to the chemosensory family. In addition, the secondary structure model of the gustatory receptor by pfam (http://pfam.xfam.org/) and HMMER v3.1b2 (http://www.hmmer.org/) were used to search the target sequences in the protein database translated from the DEG database. Finally, using the gustatory receptor C-terminal conserved sequences (hh(G/A/S) (A/S)hhTYhhhhhQF) as a template, tBLASTn searches were performed in DGE data. Using these three methods in combination, we obtained all of the gustatory receptors in BPH.

### Cloning of *NlGr* genes

The cDNA from various tissues, including head, midgut, fat body, ovary and whole body, was used as a template to clone the cDNA sequence of *NlGrs*. 3’ RACE and 5’ RACE PCR was further performed to get the complete cDNA sequences of Grs using a SMARTer^®^ RACE 5′/3′ kit (Takara Bio) according to the manufactures’ manuals. PCR products were purified using a Gel Extraction Kit (OMEGA Bio-Tek, USA), cloned into the *pEASY*^®^ Blunt vector (TRANSGEN BIOTECH, Beijing, China) and then sequenced by IGE Biotechnology, Ltd. (Guangzhou, China). Then tBLASTn searches against the NCBI database to confirm that the cloning sequences belong to the Gr family. The primers used for gene clone are listed in supplementary file (see Additional file 5: Table S5).

### Phylogenetic analysis

To investigate the expanding types of 29 BPH Grs, we selected 266 published insect Grs as the reference source. Among these 266 Grs, 68 were from *Drosophila melanogaster* (*DmGrs*), 65 were from *Bombyx mori* (*BmGrs*), 13 were from *Apis mellifera* (*AmGrs*), 58 were from *Aedes aegypti* (*AgGrs*), and 62 were from *Tribolium castaneum* (*TcasGrs*). Amino acid sequences were used for phylogenetic analysis in MEGA 6.0 [47]. A maximum-likelihood tree was calculated using the default settings and the Jones-Taylor-Tornton (JTT) with Freqs. (+F) model with partial deletions and 2000 bootstrap replications was applied in the calculation. The software TMpred (https://embnet.vital-it.ch/software/TMPRED_form.html), HMMTOP (http://www.enzim.hu/hmmtop/), and TMHMM (http://www.cbs.dtu.dk/services/ TMHMM/) were used for the prediction of transmembrane domains.

### Expression profiles of *NlGr* genes

Total RNA was collected from the ovaries, midgut, fat body, legs and heads of third-day brachypterous female adults for tissue-specific expression profiles. RNA from the first to fifth instar nymph stages and brachypterous female adults (1–5 days old) were isolated for developmental expression profiles. The total RNA was extracted based on the method described above. For the gene transcription assay, 1 μg RNA was used for first-strand cDNA synthesis using a PrimeScript™ 1st Strand cDNA Synthesis Kit (Takara Bio, Kyoto, Japan). The mRNA level was detected by quantitative real-time PCR (qRT-PCR), the primers are listed in supplementary file (see Additional file 5: Table S5).

### Prediction and verification of alternative splicing

To identify alternative splicing events in BPH, we generated a relatively complete set of transcripts based on all available transcriptomes with different developmental stages, sexes and phenotypes (see Additional file 6: Table S6), following a computational pipeline presented in supplementary file (see Additional file 7: Figure S1). Firstly, the reads of each RNA-seq dataset were mapped separately to the reference genome using TopHat [46]. The alignment files produced by TopHat were loaded into Cufflinks to generate a transcriptome assembly for each dataset [48]. These assembled transcripts were combined into a general transcriptome assembly using the Cuffmerge module, and the alternative splicing events were identified according to the GTF file from Cuffmerge. Considering the high false positives of the alternative splicing events predicted from single-exon genes, we removed transcripts with single exon. After identification, cDNA was used as the template to clone the predicted isoforms sequence, and then cloned the *NlGrs* into the genome. The primers used for verification are listed in supplementary file (see Additional file 8: Table S7).

### Herbivory treatments

The resistant rice varieties (IR26, IR36, IR56) and susceptible rice variety TN1 (Taichuang native 1) (used as a control) were provided by the International Rice Research Institute (IRRI, Los Banos, the Philippines). Thirty one-day old brachypterous BPH females starved for at least 3 h prior to the experiment were put on each rice variety. Five individuals were sampled for qRT-PCR at each time point of 3 h, 6 h, 24 h and 24 h post feeding on rice. There were three replications for each treatment and sampling time point. All the collected samples were placed in liquid nitrogen immediately, and then stored at − 80 °C for qRT-PCR.

### RNAi experiment and quantitative real-time PCR analysis

The dsRNA was produced using the T7 RiboMAX™ Express RNAi System (Promega, USA). For each *NlGr*, thirty newly emerged virgin females were used for the injection experiment. 50-nanoliter of dsRNA (4 μg/μl) was injected into the side of the abdomen of BPH using 3.5 Drummond needles and the NARISHIGE IM-31 (Nikon, Tokyo, Japan). Five individuals that survived for 48 and 72 h after injection were randomly collected for RNA extraction. GFP gene (DQ389577) was used as a control dsRNA. Each treatment was performed in triplicate. qRT-PCR also used a previously described method [49]. The primers used for dsRNA preparation and real-time PCR are listed in Table S5.

### Statistical analysis

For statistical analysis of the qRT-PCR results, the relative expression were calculated by 2^−△△Ct^ values as previously described [50]. The mRNA levels of target genes were normalized relative to the *β*-actin mRNA levels, and all of the results are expressed as the means + SE; the differences between two groups were analyzed using *t*-tests. The differences between multiple groups were analyzed using one-way analysis of variance followed by Duncan’s multiple range test for multiple comparisons. Differences were considered to be significant at *P* < 0.05 and to be very significant at *P* < 0.01.

## Additional files

Additional file 1: **Table S1.** Summary of *Nilaparvata lugens* transcriptome assembly. (XLSX 8 kb)

Additional file 2: **Table S2.** Screening for putative gustatory receptors of *Nilaparvata lugens* by tBLASTn. (XLSX 10 kb)

Additional file 3: **Table S3.** Prediction of potential gustatory receptors of *Nilaparvata lugens* by HMMER. (XLSX 9 kb)

Additional file 4: **Table S4.** Knockdown the expression of *NlGrs* in BPH and its effect on *NlVg* and *NlVgR* transcript accumulation. (XLSX 12 kb)

Additional file 5: **Table S5.** The primers used in this study. (XLSX 11 kb)

Additional file 6: **Table S6.** The data source for alternative splicing prediction. (XLSX 10 kb)

Additional file 7: **Figure S1.** Computational pipeline for identifying alternative splicing events in *N. lugens* from RNA-seq data. (JPG 64 kb)

Additional file 8: **Table S7.** The primers used in alternative splicing verification. (XLSX 9 kb)

## Abbreviations

AKT: Protein kinase B
AMPK: Adenosine 5′-monophosphate (AMP)-activated protein kinase
BPH: The brown planthopper
cDNA: Complementary DNA
DGE: Digital gene expression
dsRNA: Double-stranded RNA
Gr: Gustatory receptor
GTF: Gene transfer format
JTT: Jones-Taylor-Tornton
NCBI: National Center for Biotechnology Information
Or: Odorant receptor
ORF: Open reading frame
PCR: Polymerase chain reaction
qRT-PCR: Quantitative real time RT-PCR
RACE: Rapid-amplification of cDNA ends
RNAi: RNA interference
SE: Standard error
tBLASTn: Translated basic local alignment search tool
Vg: Vitellogenin
VgR: Vitellogenin receptor
