# Supplementary figures and images for: Identification of putative fecundity-related gustatory receptor genes in the brown planthopper Nilaparvata lugens
Source: BMC Genomics. 2018 Dec 27;19:970. doi: 10.1186/s12864-018-5391-5 (PMC6307266; doi:10.1186/s12864-018-5391-5)

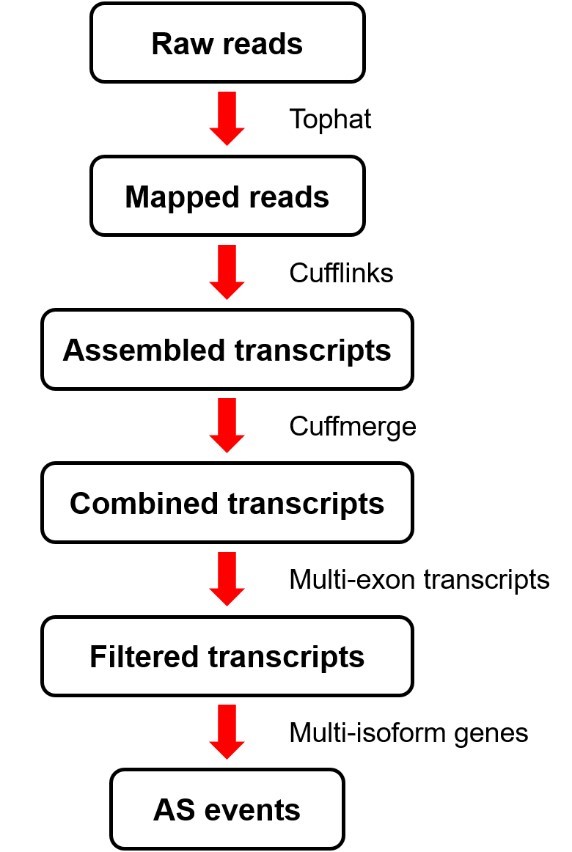

Supplement: Supplementary file 7 — Figure S1. Computational pipeline for identifying alternative splicing events in N. lugens from RNA-seq data. (JPG 64 kb) [file 12864_2018_5391_MOESM7_ESM.jpg]
